# Cooccurrence of Darier's Disease and Epilepsy: A Pediatric Case Report and Review of the Literature

**DOI:** 10.1155/2014/831398

**Published:** 2014-09-01

**Authors:** Tamer Celik, Umit Celik, Cigdem Donmezer, Mustafa Komur, Orkun Tolunay, Pelin Demirtürk

**Affiliations:** ^1^Pediatric Neurology, Adana Numune Research and Training Hospital, Adana, Turkey; ^2^Pediatrics, Adana Numune Research and Training Hospital, Adana, Turkey; ^3^Pathology Department, Adana Numune Research and Training Hospital, Adana, Turkey

## Abstract

Darier's disease is a skin disorder characterized by multiple eruptions of hyperkeratosis or crusted papules at seborrheic areas with histologic acantholysis and dyskeratosis. It is caused by mutations in a single gene, being *ATP2A2* and that is expressed in the skin and brain. The cooccurrence of various neurologic and psychiatric diseases with Darier's disease has been reported frequently in literature. They include mood disorders, epilepsy, encephalopathy, and schizophrenia. In this study, we report a pediatric case with the cooccurrence of Darier's disease and epilepsy. We also revised current English literature on this topic.

## 1. Introduction

Darier's disease (DD, Lutz-Darier-White disease, keratosis follicularis; MIM 124200) is a skin disorder characterized by multiple eruptions of hyperkeratotic or crusted papules and plaques at seborrheic areas, palmoplantar pits, and nail abnormalities [1]. The prevalence of the disease is estimated to be 1 in 50000 people [2]. DD is autosomal, dominantly inherited with high penetrance, although phenotypic expression is variable [2]. The linear form of DD is not inherited; it rather occurs as a result of somatic mutations. No family history is common and it represented a third of cases reported in a study [3].

DD is caused by mutations in the* ATP2A2* gene, which maps to chromosome 12q23-q24.1 and encodes the sarcoplasmic/endoplasmic reticulum calcium ATPase isoform 2 (SERCA2), a calcium pump located in the endoplasmic reticulum membrane [4, 5]. Darier's disease is caused by reduction in the SERCA2b function leading to abnormal intracellular Ca+2 signalling and abnormal organization or maturation of complexes responsible for cell adhesion [6].

DD is presenting in adolescence or adulthood with the onset of multiple focal keratotic skin lesions [4, 5]. Skin lesions may be severe, with widespread itchy malodorous crusted plaques, painful erosions, blistering, and mucosal lesions. Sun, heat, and sweating exacerbate the disease. Males and females are affected equally by the disease. Several variants of cutaneous lesions have been reported in literature [4]. Bullous, erosive, vegetative form, familial haemorrhagic variant, and hypopigmented macules were seen in DD patients. Seborrheic dermatitis, Hailey-Hailey disease, and transient acantholyticdermatosis should be assessed in differential diagnosis [7]. The cooccurrence of various neurologic and psychiatric diseases including mood disorders, mental retardation, and schizophrenia has been reported with DD [8, 9]. However, there are only a few reports that indicate the cooccurrence of epilepsy and DD.

In this study, we report a pediatric case with cooccurrence of DD and epilepsy. We also revised the current English literature existing on this topic.

## 2. Case Report

**Figure 1:**
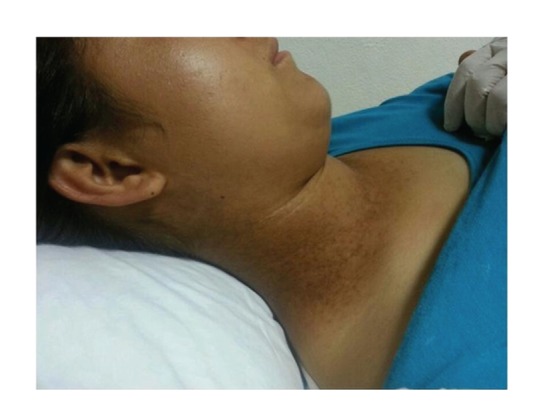
Hyperkeratotic dark papules on the neck.

**Figure 2:**
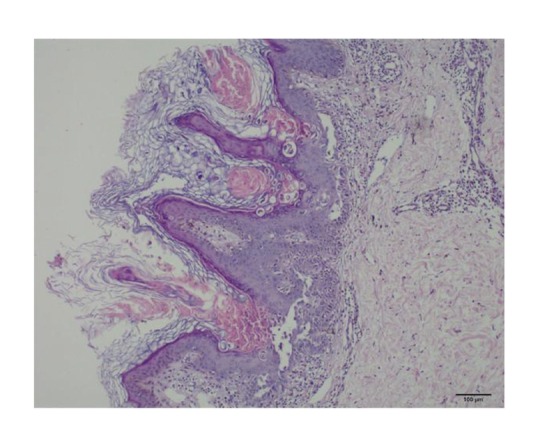
Skin biopsy showing hyperkeratosis, dyskeratosis, papillomatosis, suprabasal acantholysis, and dermal chronic inflammatory infiltrate (H&E ×100).

A sixteen-year-old female patient was referred to the Pediatric Neurology Department because of seizures. The history of the patient revealed ordinary vaginal birth of 3 kg and no problems in neonatal and early childhood but some learning difficulties and low success in the school at the time the patient started primary school. There is no similar disease history in the family. Within the last year, she also had a history of 3 generalized tonic-clonic seizures that lasted for 3-4 minutes. However, it was learned that she has never applied to a physician. According to the medical history, she had rash in the neck and behind the ears that had begun two years ago. Although the skin lesions were sometimes itchy, heat or sweating did not worsen them. In her examination, hyperkeratotic papules in 0.3 × 0.5 cm dimensions were detected, being hard with palpation, presented on a postauricular area, and ranging in length from neck and covering the scalp and ears (Figure 1). Neurologic and other system examinations, biochemical tests and complete blood cell count, electrocardiogram, cerebral magnetic resonance imaging, and finally awake and sleep electroencephalogram were normal. The patient consulted a dermatologist and punch biopsy of the skin was taken. On histopathological examination; hyperkeratosis, dyskeratosis, papillomatosis, suprabasal acantholysis, and dermal chronic inflammatory infiltrate were seen and Darier's disease was confirmed (Figure 2).

## 3. Discussion

DD appears in association with an increased prevalence of neuropsychiatric disorders including mental retardation, epilepsy, schizophrenia, bipolar disorder, and sociopathic behaviour [4, 10, 11]. The majority of DD patients have no neuropsychiatric problems. The reported prevalence rates and types of neuropsychiatric features in studies vary greatly. It is not known if neuropsychiatric symptoms are a psychological reaction to having a skin disease or a direct consequence of mutations in the* ATP2A2* gene [12]. DD gene has pleiotropic effects in skin and brain [5]. These tissues share a common ectodermal origin and intracellular calcium signalling in neurons is involved in neuronal excitability and neurotransmission [13]. SERCA2 is a calcium pump of the endoplasmic reticulum transporting calcium from the cytosol to the lumen of endoplasmic reticulum [12].* ATP2A2* mutations lead to loss of calcium transport by SERCA2 resulting in decreased endoplasmic reticulum calcium concentration in Darier's keratinocytes [12]. The role of SERCA2 in neurological development or function remains highly plausible since the gene is widely expressed in the brain [14]. One study suggests that a susceptibility locus for bipolar disorder cosegregates with the keratosis follicularis region, but it is distinct from the keratosis follicularis-causing mutation [15]. Missense, nonsense, frameshift, and splicing mutations in the ATPA2 gene of families with DD have been described [1]. Up to date, more than 120 familial and sporadic mutations in* ATP2A2* have been identified in DD patients but attempts at identifying genotype-phenotype correlation have not been successful [7]. Ruiz-Perez et al. reported that variant cutaneous phenotypes associated with missense mutations. Contrarily, they also reported that neuropsychiatric features are not associated with a spesific type of mutation but rather depend on concomitant genetic and environmental factors [16]. On the other hand, Jacobsen et al. reported that missense mutations in* ATP2A2* correlate with the presence of neuropsychiatric phenotypes and more specifically that the ATP binding domain may have relevance in mood disorders [5]. Comparison of molecular data and association with neuropsychiatric disorders do not reveal an obvious genotype-phenotype correlation in Ringpfeil et al.'s study [17]. In a study in North African population, no obvious phenotype-genotype correlation was established [18].

Our patient was referred to the Pediatric Neurology Department for evaluation of her seizures. There, we detected skin lesions (Figure 1). Seborrheic dermatitis and transient acantholysis dermatosis in differential diagnosis were excluded. By evaluation of the clinical characteristics and following biopsy, DD was diagnosed. Here, genetic analysis was not performed. The patient had epileptic and also learning problems. While the reported lifetime prevalence rates of epilepsy in the general population are variable, with the percentage 1.3%, the overall prevalence of epilepsy is 30–42.9 in 1000 DD patients [4, 19, 20]. A higher prevalence of epilepsy in individuals with DD was seen compared with that in general population [4, 20]. In the study of Gordon-Smith et al., mood disorders (50%), specifically major depression (30%), bipolar disorder (4%), suicide attempts (13%), suicidal thoughts (31%), and epilepsy (3%), in 100 DD patients were reported [20]. The association does not appear as a specific subtype of epilepsy. In some DD cases with epilepsy, cerebral atrophy was reported in computed tomography of the brain [21] but our patient's MRI findings were normal.

DD patients may show moderate learning and behavioral problems [1]. Learning difficulties are related not only to the IQ level but also to many factors such as social isolation, and low social condition may contribute to this situation. In the study of Gordon-Smith et al., the IQ level of DD patients was found to be indifferent to the general population [20]. However, in a study limited to just a few cases, the IQ of 2 of the 5 patients was detected to be below the average [10]. Mental retardation has been reported in some families. In a British report, 5% of 163 patients were mentally retarded and the same proportion was epileptic [4]. A study conducted in Denmark provides us with the information that seven of the 37 patients were mentally subnormal and nine others were destitute [22].

In conclusion, both pediatric neurologists and dermatologists should be aware of a possible cooccurrence of Darier's disease and epilepsy. The recognition and treatment of epilepsy will prevent complications that may develop and can also improve the patient's life quality.
